# Aromatic Amino Acid-Derived Compounds Induce Morphological Changes and Modulate the Cell Growth of Wine Yeast Species

**DOI:** 10.3389/fmicb.2018.00670

**Published:** 2018-04-11

**Authors:** Beatriz González, Jennifer Vázquez, Paul J. Cullen, Albert Mas, Gemma Beltran, María-Jesús Torija

**Affiliations:** ^1^Departament de Bioquímica i Biotecnologia, Universitat Rovira i Virgili, Tarragona, Spain; ^2^Department of Biological Sciences, University at Buffalo, Buffalo, NY, United States

**Keywords:** aromatic alcohols, serotonin, tryptamine, quorum sensing, pseudohyphal growth, non-*Saccharomyces*, invasive growth

## Abstract

Yeasts secrete a large diversity of compounds during alcoholic fermentation, which affect growth rates and developmental processes, like filamentous growth. Several compounds are produced during aromatic amino acid metabolism, including aromatic alcohols, serotonin, melatonin, and tryptamine. We evaluated the effects of these compounds on growth parameters in 16 different wine yeasts, including non-*Saccharomyces* wine strains, for which the effects of these compounds have not been well-defined. Serotonin, tryptamine, and tryptophol negatively influenced yeast growth, whereas phenylethanol and tyrosol specifically affected non-*Saccharomyces* strains. The effects of the aromatic alcohols were observed at concentrations commonly found in wines, suggesting a possible role in microbial interaction during wine fermentation. Additionally, we demonstrated that aromatic alcohols and ethanol are able to affect invasive and pseudohyphal growth in a manner dependent on nutrient availability. Some of these compounds showed strain-specific effects. These findings add to the understanding of the fermentation process and illustrate the diversity of metabolic communication that may occur among related species during metabolic processes.

## Introduction

Wine is produced by alcoholic fermentation, in which grape sugars are metabolized into ethanol by yeast. During grape ripening, the surfaces of berries are primarily colonized by non-*Saccharomyces* yeast, such as *Hanseniaspora*, *Starmerella* (sym *Candida*), *Hansenula*, or *Metschnikowia*. Microorganisms belonging to the *Saccharomyces* genus are present in low abundance and are difficult to detect in initial must (Ribéreau-Gayon et al., 2006). For this reason, during spontaneous fermentation, non-*Saccharomyces* yeasts are responsible for initiating alcoholic fermentation and are then out-competed by *S. cerevisiae* throughout fermentation (Heard and Fleet, 1988; Fleet, 2003; Ribéreau-Gayon et al., 2006). Traditionally, the low ethanol tolerance and competitiveness of non-*Saccharomyces* yeasts compared to *Saccharomyces* species (Ribéreau-Gayon et al., 2006) has resulted in a lack of interest in these yeast species for many years. However, recently, the importance of non-*Saccharomyces* strains in alcoholic fermentation has become appreciated, particularly in terms of their contribution to wine aroma, during the early steps of fermentation. Indeed, these species have been reported to impact, sometimes positively, winemaking via the production of high amounts of aromatic compounds, such as aromatic alcohols, ethyl esters, and acetate esters (Romano et al., 2003; García et al., 2010; Jolly et al., 2014; Belda et al., 2017). Furthermore, these strains appear to be present throughout much of the fermentation process, although this finding has been neglected because such strains are difficult to culture (Millet and Lonvaud-Funel, 2000; Wang et al., 2015a, 2016).

*Saccharomyces cerevisiae* is a unicellular fungi that reproduce asexually by budding and is able to undergo filamentous growth to scavenge for nutrients (Wendland and Philippsen, 2001; Verstrepen and Klis, 2006; Cullen and Sprague, 2012). Filamentous growth includes morphological changes that involve the global reorganization of cellular processes to produce a new cell type. Cells alter their budding pattern, becoming more elongated and remaining attached to each other through the formation of pseudohyphae. Moreover, under certain conditions, yeast cells penetrate surfaces through a process known as invasive growth (Roberts and Fink, 1994). Although much of the genetic characterization of this response has been performed in *S. cerevisiae* strains on the Σ1278b background (Gimeno et al., 1992; Cullen and Sprague, 2000), the response has also been studied in many strains and genera (Gimeno and Fink, 1994; San-blas et al., 1997; Lo and Dranginis, 1998). For example, the human pathogen *Candida albicans* (Hornby et al., 2001; Chen et al., 2004; Biswas et al., 2007; Kruppa, 2009) undergoes pseudohyphal and hyphal growth (pathogenic form), which confers the ability to infect human tissues (Lo et al., 1997; Leberer et al., 2001; Rocha et al., 2001). Filamentous growth in yeasts has been reported to occur in response to cell density and several molecules, such as aromatic alcohols and ethanol, have been identified as stimuli that induce these morphological changes (Gimeno et al., 1992; Dickinson, 1996; Lorenz et al., 2000; González et al., 2017). Indeed, aromatic alcohols, tyrosol (TyrOH), tryptophol (TrpOH), and phenylethanol (PheOH), which are derived from the amino acids tyrosine, tryptophan, and phenylalanine, respectively, have been suggested to act as quorum sensing molecules (QSMs) in yeasts, regulating cell density and evoking morphogenetic transitions (Chen et al., 2004; Chen and Fink, 2006). Moreover, nitrogen limitation results in the increased production of aromatic alcohols, leading to elevated filamentous growth in *S. cerevisiae*. In this species, PheOH and TrpOH act as inducers of morphogenesis, while TyrOH has no detectable effects (Chen and Fink, 2006). However, in *C. albicans*, these alcohols exhibit the opposite behavior: TyrOH promotes pseudohyphal growth, whereas PheOH and TrpOH inhibit it. The finding that different aromatic alcohols exert different responses on morphogenesis depending on the yeast species implicates these molecules as inducers of species-specific effects (Chen and Fink, 2006). In a recent study, González et al. (2017) showed that ethanol specifically induced filamentous growth under nitrogen-limiting conditions, whereas aromatic alcohols did not. Thus, environmental conditions impact the efficacy of these compounds. Non-*Saccharomyces* yeasts, such as *Hanseniaspora uvarum*, *Pichia kudriavzevii*, and *Pichia fabianii*, undergo filamentous growth under nutrient-limited conditions (nitrogen or carbon) or in the presence of other stress factors (Pu et al., 2014; van Rijswijck et al., 2015), but the roles of these alcohols have not been extensively explored.

During alcoholic fermentation, yeast synthesizes compounds that, depending on the concentration, can be inhibitory to their own growth or the growth of other yeast species. A primary example is ethanol, which is a potent inhibitory compound for growth. Other metabolites, such as short-to-medium-chain fatty acids (e.g., acetic, hexanoic, octanoic, and decanoic acids) and yeast killer toxins, also inhibit growth and even induce the death of certain yeast species, including strains of *S. cerevisiae* (Pérez et al., 2001). Recently, interactions between species were shown to be impacted by the secretion of compounds by yeast during alcoholic fermentation (Ciani and Comitini, 2015; Wang et al., 2015b; Albergaria and Arneborg, 2016). To our knowledge, there have been no studies investigating the effects of aromatic alcohols or other QSMs synthesized during alcoholic fermentation on the growth and vitality of wine yeasts. Moreover, the effects of aromatic alcohols on the filamentous growth of non-*Saccharomyces* wine yeast species have not been explored. The investigation of these areas might help to unravel the possible roles of QSMs in the interactions between yeasts during alcoholic fermentation. Moreover, direct microbial interactions (i.e., through physical contact) are reportedly involved in the growth inhibition of non-*Saccharomyces* yeast, although such mechanisms are dependent on cell density, when cultures are competing for space (Nissen et al., 2003, 2004; Pérez-Nevado et al., 2006; Renault et al., 2013).

Additionally, through tryptophan metabolism, yeasts also produce other metabolites that are related to indoles, such as serotonin, melatonin, or tryptamine. Serotonin and melatonin are of special relevance for their bioactivity in higher organisms, including humans. Rodriguez-Naranjo et al. (2012) demonstrated that melatonin is produced during alcoholic fermentation by yeast, and different strains and species synthesize this compound at different concentrations. The role of melatonin in yeasts is still unclear, although a recent paper showed that the compound demonstrated possible antioxidant activity in response to oxidative damage by hydrogen peroxide in *S. cerevisiae* (Vázquez et al., 2017). On the other hand, tryptamine has also been detected in red wines at mg/L concentrations after malolactic fermentation (Wang et al., 2014). Serotonin appears to exert antifungal activity against *Candida* and *Aspergillus* spp. *in vitro* (Lass-Flörl et al., 2002, 2003).

Thus, the objective of this study was to evaluate the effects of different compounds derived from aromatic amino acid metabolism and produced during alcoholic fermentation on the growth and physiology of different wine yeast species. We first described an analysis of the growth parameters of different yeast strains and species in the presence of increasing concentrations of specific compounds of interest. Then, the effects of aromatic alcohols and ethanol, which are well-known morphogenesis inducers in *S. cerevisiae*, were examined for their impact on the filamentous growth of different non-*Saccharomyces* wine species.

## Materials and Methods

### Strains and Growth Media

Eight strains from *Saccharomyces* species and two strains from four species of non-*Saccharomyces* yeast were used in the study. The *S. cerevisiae* strains included the laboratory strain Σ1278b, the wine strains SB (Marullo et al., 2007), QA23, T73, P5, and P24 (Lallemand, Canada), the animal nutrition strain Sc20 and the hybrid *S. kudriavzevii/S. cerevisiae* Vin7 (Oenobrands SAS, France) (Borneman et al., 2012). The non-*Saccharomyces* yeasts were *Starmerella bacillaris* (sym. *Candida zemplinina*) (Cz4-CECT13129, Cz11), *H. uvarum* (Hu4-CECT13130, Hu11), *Metschnikowia pulcherrima* (Mpp-CECT 13131, FLAVIA), and *Torulaspora delbrueckii* (Tdp-CECT 13135, BIODIVA). FLAVIA and BIODIVA are commercial strains (Lallemand, Canada) whereas the other non-*Saccharomyces* strains were isolated from grapes/wine media (Padilla et al., 2016). Yeasts were typically grown on YPD [2% (w/v) peptone, 1% (w/v) yeast extract, 2% (w/v) glucose, and 2% (w/v) agar] at 28°C.

### Effects on Yeast Growth

Yeasts were pre-cultured for 48 h on minimal medium [(MM) 1x Yeast Nitrogen Base (YNB) without (w/o) amino acids (aa) or ammonium, 2% (w/v) glucose, and 10 mM (NH_4_)_2_SO_4_ (280 mgN/L)] at 28°C and then inoculated into each medium, adjusting the initial optical density (OD_600nm_) to 0.2. To evaluate the effects of nitrogen concentration, yeasts were grown on MM and on low nitrogen medium [(LNM) 1x YNB w/o aa or ammonium, 2% (w/v) glucose, and 1 mM (NH_4_)_2_SO_4_ (28 mgN/L)]. Media were supplemented with increasing concentrations of melatonin (Mel), tryptamine (Trpm), serotonin (Ser), tyrosol (TyrOH), phenylethanol (PheOH), and tryptophol (TrpOH), ranging from 50 to 1000 mg/L. All assays were performed using a POLARstar Omega microplate reader (BMG LABTECH, Germany) and were performed in triplicate at 28°C for 48 h. Microplate wells were filled with 250 μL of inoculated media. A control well-containing medium without inoculum was used to determine the background signal. Measurements were taken every 30 min after pre-shaking the microplate for 30 s at 500 rpm. For each growth curve, the variables generation time (GT) and maximal growth (OD max) were calculated according to Warringer and Blomberg (2003). Briefly, for the GT determination, a slope was calculated between every second consecutive measurement for the whole growth curve (OD values were previously log_10_ transformed). Of the seven highest slopes, the highest two were discarded, and the mean for the following five was defined as maximum division rate. The GT was obtained dividing the log_10_ 2 by the maximum division rate. The lag phase was calculated using the program GrowthRates (Hall et al., 2014).

#### Statistical Data Processing

All experiments were performed in triplicate. The data was subjected to one-way analysis of variance (ANOVA), and Tukey’s *post hoc* test (XLSTAT Software) was used to evaluate significant differences between the control condition (no addition) and the addition of each compound. The results were considered statistically significant at *p* < 0.05. For each compound, relative values were calculated using the condition in the absence of added compound (0 mg/L) as a control [(condition-control)/control]. To better understand the interactions between the calculated parameters and their effects on yeast growth, principal component analysis (PCA) was performed using XLSTAT Software at a concentration of 1000 mg/L for each compound and under both nitrogen conditions (MM and LNM) for all strains tested.

### Filamentous Growth Assays

#### Yeast Strains, Media, and Growth Conditions

For the filamentous growth assay, two strains of each non-*Saccharomyces* species were tested, using the strain QA23 (*S. cerevisiae*) as a control (González et al., 2017). Yeasts were grown on minimal medium [MM – 1x YNB w/o aa or ammonium, 2% (w/v) glucose, and 10 mM (NH_4_)_2_SO_4_] with agitation (120 rpm) for 16 h at 28°C before seeding on plates for filamentation analysis. To evaluate invasive and pseudohyphal growth, three different media were used, with variations in glucose and nitrogen concentrations: SAD – synthetic medium [1x YNB w/o aa or ammonium, 2% (w/v) glucose, and 37 mM (NH_4_)_2_SO_4_ and 2% (w/v) agar], SALG – synthetic medium with low glucose [1x YNB w/o aa or ammonium, 0.5% (w/v) glucose, and 37 mM (NH_4_)_2_SO_4_ and 2% (w/v) agar] (González et al., 2017), and SLAD – synthetic low-ammonium dextrose medium [SLAD – 1x YNB w/o aa or ammonium, 2% (w/v) glucose, and 50 μM (NH_4_)_2_SO_4_ and 2% (w/v) agar]. To test the effects of aromatic alcohols, the above media were supplemented with 500 μM of TyrOH (6,90 mg/L), TrpOH (8,06 mg/L) or PheOH (6,10 mg/L) or 2% (v/v) ethanol. Those concentrations were chosen according our previous studies (González et al., 2017).

#### Invasive and Pseudohyphal Growth Assays

Cells pre-grown in MM for 16 h were harvested by centrifugation, washed once in sterile water, and adjusted to an OD_600nm_ of 2.0. Subsequently, 10 μl of cells were spotted in triplicate on semisolid agar media. Plates were incubated at 30°C for 3, 5, and 7 days depending on the experiment. Invasive growth was determined in a plate washing assay (Roberts and Fink, 1994). Colonies were photographed before and after the plates were washed in a stream of water, after which the colonies were rubbed from the surface with a gloved finger. ImageJ software^1^ was used to quantitative invasive growth in the plate-washing assay. The background intensity was determined for each spot and subtracted from the densitometry of the invasive area. Densitometry analysis was performed on invasive patches over multiple days. The data was subjected to one-way ANOVA and Tukey’s *post hoc* test (XLSTAT Software) was used to evaluate significant differences on invasion intensity between media. The results were considered statistically significant at *p* < 0.05. The examination of pseudohyphae was determined as described by Gimeno et al. (1992). Before washing the plates, the colony periphery was observed and photographed each day under microscopy (Raman FT-IR).

## Results

### Effects of the Presence of Aromatic Amino Acid-Derived Compounds on Yeast Growth

**FIGURE 1:**
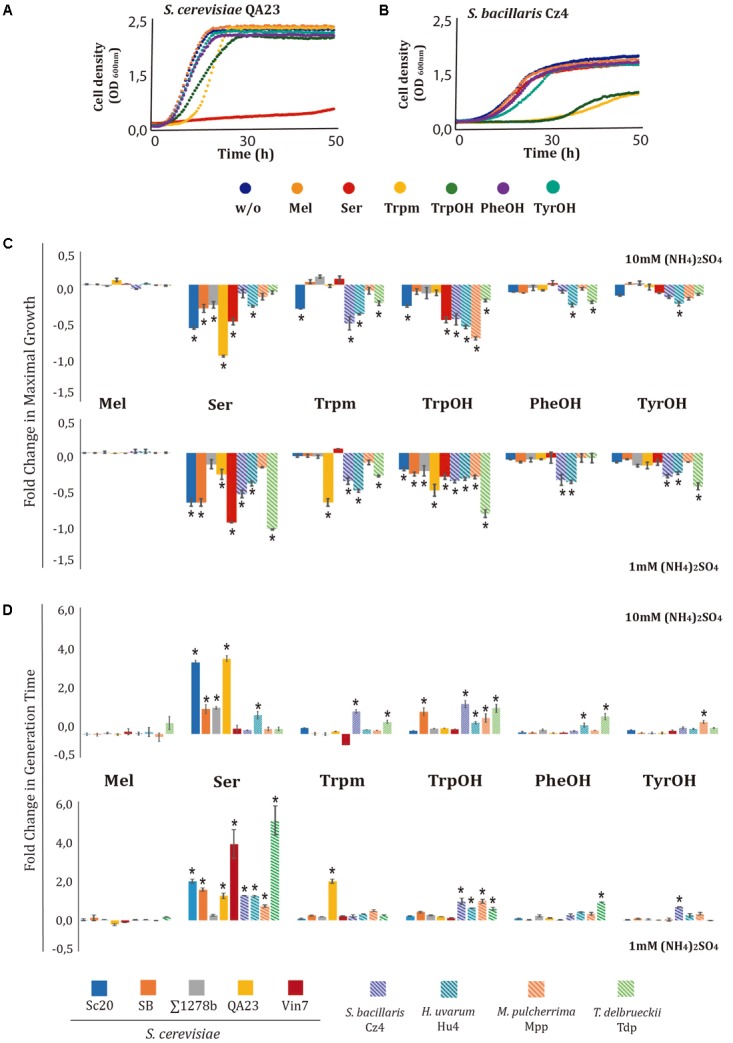
Effects of aromatic amino acid-derived compounds on the growth of wine yeast species at different nitrogen concentrations. The effects of Mel, Ser, Trpm, TrpOH, PheOH, and TyrOH on the growth of four strains of *S. cerevisiae* and four of non-*Saccharomyces* were determined. Yeast were grown for 48 h at 28°C in minimal medium with two different nitrogen concentrations [10 mM or 1 mM (NH_4_)_2_SO_4_] and supplemented with 1000 mg/L of each compound. Non-supplemented cultures were used as controls. Experiments were carried out in triplicate. Growth curves of *S. cerevisiae* QA23 **(A)** and *S. bacillaris* Cz4 **(B)**, with the different compounds added at 10 mM (NH_4_)_2_SO_4_ medium are shown. For each nitrogen condition and compound, maximal growth **(C)**, and generation time **(D)** was calculated. The fold-change for each growth parameter was determined in relation to its control condition. Statistical analysis was performed using Tukey’s test by comparing the effects of each compound in the different strains; asterisk denotes a *p*-value < 0.05.

To evaluate the effects of amino acid-derived compounds on yeast growth, five strains of *S. cerevisiae* and one strain of each non*-Saccharomyces* species were grown in the presence of 1000 mg/L of Mel, Ser, Trpm, TyrOH, PheOH, or TrpOH. As these molecules are derived from nitrogen metabolism, and QSMs are produced during nutrient limitation, we tested their effects under two different nitrogen conditions: 1 and 10 mM (NH_4_)_2_SO_4_ (**Figure 1**). As an example, the growth curves obtained with *S. cerevisiae* QA23 (**Figure 1A**) and *S. bacillaris* Cz4 (**Figure 1B**) in the presence of 1000 mg/L of the different compounds and 10 mM (NH_4_)_2_SO_4_ are shown. In the QA23 strain, Ser completely inhibited cell growth. In addition to this dramatic phenotype, other subtle phenotypes were observed. TrpOH caused a reduction in growth rate and maximal growth, and Trpm increased the lag phase. The other compounds tested did not significantly affect the growth profile. In comparison, the growth of strain Cz4 was reduced by TrpOH and Trpm, but not by the other compounds. Therefore, different compounds cause the growth inhibition of different species.

The relative values of OD max (**Figure 1C**) and GT (**Figure 1D**) were calculated for each compound, using the condition without addition as a control (absolute values can be found in Supplementary Table S1). Overall, the addition of these compounds (with the exception of Mel) exerted negative impacts on the maximal growth obtained for most of the tested strains (**Figure 1C**). Ser decreased the OD max in all yeast species, particularly under low nitrogen conditions, while Trpm and aromatic alcohols had a major impact in non*-Saccharomyces* strains under both nitrogen conditions. On the other hand, Ser caused growth reduction in all strains, increasing their GT (**Figure 1D**). In general, this increase was significant for *Saccharomyces* strains under both nitrogen conditions but only under low nitrogen conditions for most non-*Saccharomyces* strains. Increases in GT were also observed when the medium was supplemented with TrpOH in all the non-S*accharomyces* strains under both nitrogen conditions. The other two aromatic alcohols, PheOH and TyrOH, exerted no effects in *Saccharomyces* strains, and at 1 mM, among non-*Saccharomyces* strains, only the Tdp strain was affected by PheOH, and *S. bacillaris* by TyrOH. In general, the relative OD max or GT presented a similar trend under both nitrogen conditions; the most relevant differences consisted of greater effects from Ser in the non-*Saccharomyces* strains under low nitrogen concentration. The effects of these compounds were impacted by exogenous nitrogen levels, although in a strain-dependent manner. The impact of ethanol on yeast growth was also analyzed, but no significant differences were observed at 1000 mg/L for any of the yeast species studied (data not shown). Based on these results, at high nitrogen concentration *Saccharomyces* and non-*Saccharomyces* strains clustered into two different groups in a PCA (Supplementary Figure S1A), primarily attributable to the higher reduction in the OD max on non-*Saccharomyces* strains due to the presence of aromatic alcohols and Trpm. Under low nitrogen conditions (Supplementary Figure S1B), all strains of *Saccharomyces* were included in the same cluster, but non-*Saccharomyces* strains were plotted into two different groups because *T. delbrueckii* clustered separately from the other non-*Saccharomyce*s species, because of their higher GT in PheOH.

### Effects of the Concentrations of Aromatic Amino Acid-Derived Compounds on Yeast Growth

According to our previous results, the effects of certain aromatic amino acid-derived compounds were slightly greater under low nitrogen conditions than under high nitrogen conditions. For this reason, we investigated how the increasing concentrations of these compounds (from 50 to 1000 mg/L) affect the growth of a larger collection of wine yeast in nitrogen-limiting conditions (absolute values of GT and maximal growth obtained for each strain and condition can be found in Supplementary Tables S2, S3).

**FIGURE 2:**
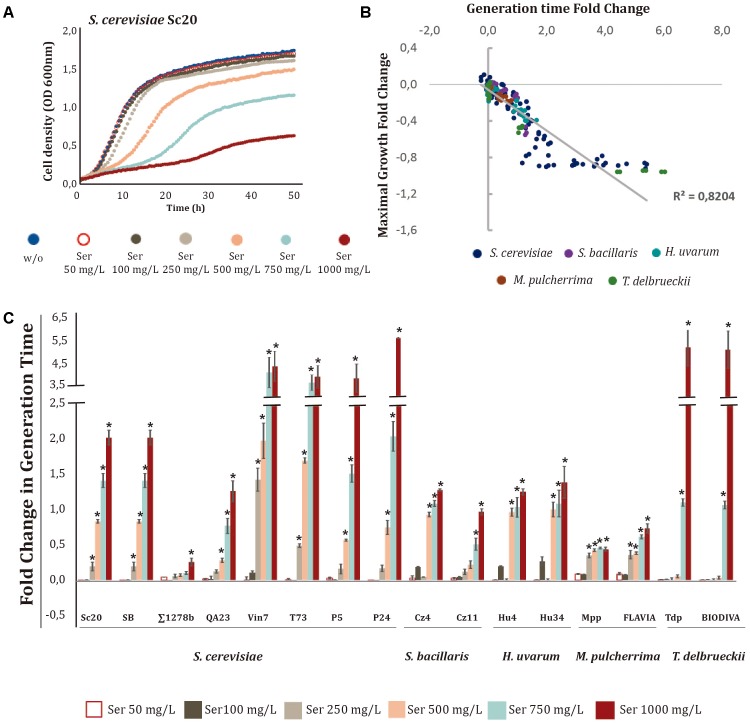
Effects of increasing serotonin (Ser) concentrations on yeast growth. Ser was added to minimal medium [1 mM (NH_4_)_2_SO_4_] at increasing concentrations (50, 100, 250, 500, 750, and 1000 mg/L). **(A)** Growth curves obtained with *S. cerevisiae* Sc20. **(B)** Correlation between the generation time and maximal growth fold-changes obtained with different yeast species. **(C)** Generation time fold-change for each strain at different Ser concentrations. Statistical analysis was performed, using Tukey’s test and comparing the effects of Ser concentrations in each strain; asterisk denotes a *p*-value < 0.05. The fold-change for each growth parameter was determined in relation to the control (no-supplemented condition, w/o).

**FIGURE 3:**
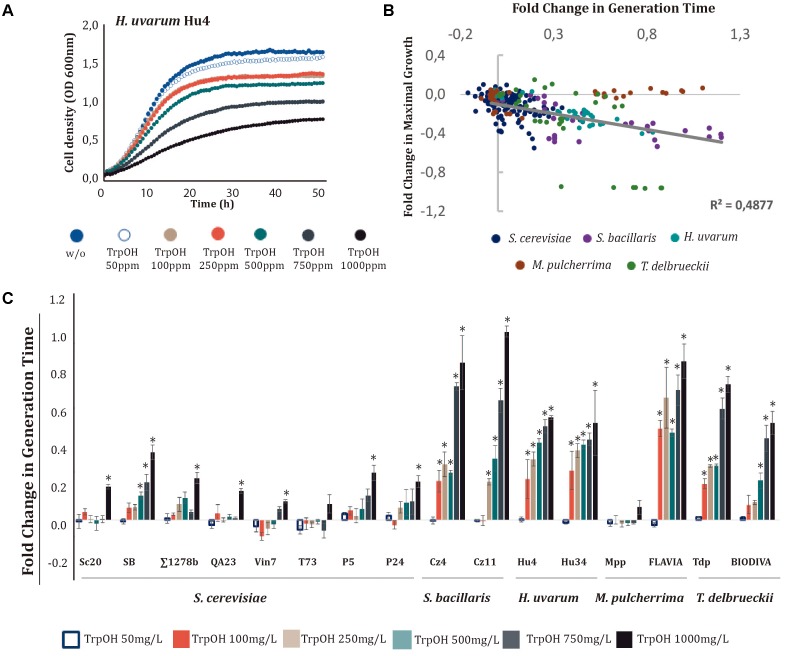
Effects of increasing tryptophol (TrpOH) concentrations on yeast growth. TrpOH was added to minimal medium [1 mM (NH_4_)_2_SO_4_] at increasing concentrations (50, 100, 250, 500, 750, and 1000 mg/L). **(A)** Growth curves obtained with *H. uvarum* Hu4. **(B)** Correlation between the generation time and maximal growth fold-changes obtained with different yeast species. **(C)** Generation time fold-change for each strain at different TrpOH concentrations. Statistical analysis was performed using the Tukey’s test and comparing the effects of TrpOH concentrations in each strain; asterisk denotes a *p*-value < 0.05. The fold-change for each growth parameter was determined in relation to the control (no-supplemented condition, w/o).

**FIGURE 4:**
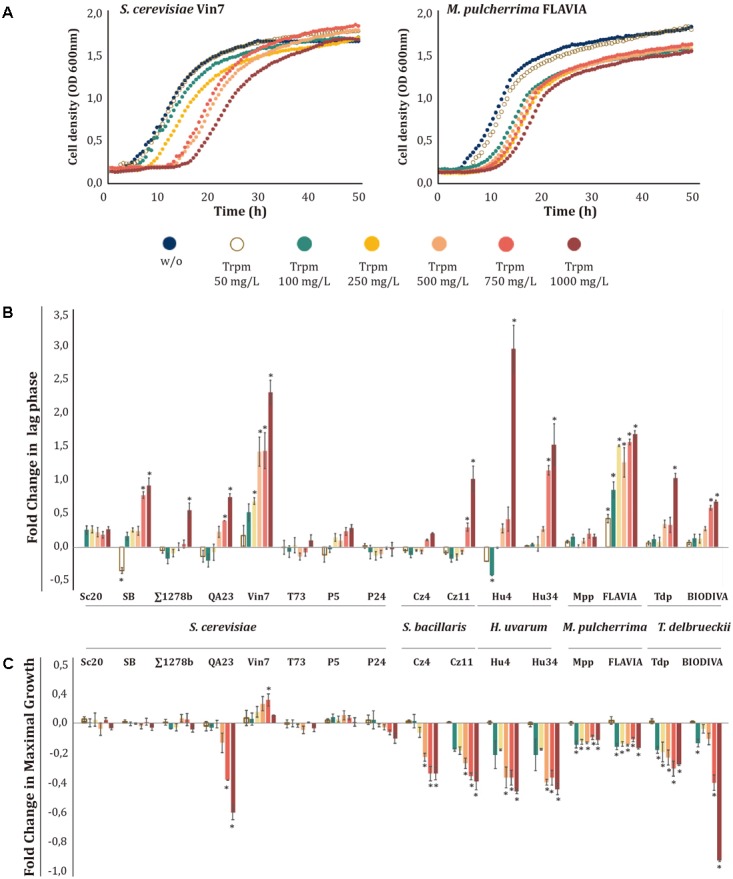
Effects of increasing tryptamine (Trpm) concentrations on yeast growth. Trpm was added to minimal medium [1 mM (NH_4_)_2_SO_4_] at increasing concentrations (50, 100, 250, 500, 750, and 1000 mg/L). **(A)** Growth curves obtained with *S. cerevisiae* Vin7 and *M. pulcherrima* FLAVIA. **(B)** Lag phase fold-change for each strain at different Trpm concentrations. **(C)** Maximal growth fold-change for each strain at different Trpm concentrations. Statistical analysis was performed comparing the effects of Trpm concentrations in each strain, using Tukey’s test statistical method; asterisk denotes a *p*-value < 0.05. The fold-change for each growth parameter was determined in relation to the control (no-supplemented condition, w/o).

When different concentrations of the metabolites were tested, we observed again that Ser (**Figure 2** and Supplementary Figure S2), TrpOH (**Figure 3** and Supplementary Figure S2), and Trpm (**Figure 4** and Supplementary Figure S2) exerted higher impacts on the cell growth of yeast strains and in some cases in the lag phase. On the other hand, TyrOH and PheOH only affected to the growth of non-*Saccharomyce*s strains (Supplementary Figure S3), even at low concentrations (50 mg/L) in the case of PheOH.

The effects of different Ser concentrations on the *S. cerevisiae* Sc20 strain are shown as an example (**Figure 2A**). Clear inhibition of yeast growth was observed at concentrations of Ser above 500 mg/L, increasing GT and decreasing the OD max. Interestingly, GT and OD max values obtained in the presence of Ser were strongly correlated (*R*^2^ 0.8204), indicating that this compound influenced both growth parameters for most strains (**Figure 2B**). As shown in **Figure 3C**, all wine strains were affected by the presence of Ser in the medium, and the increase in GT was directly proportional to the Ser concentration, illustrating a dose-dependent effect. Instead, the laboratory strain Σ1278b was barely affected by this compound. Most *S. cerevisiae* strains showed growth inhibition starting from 250 mg/L, primarily in Vin7, T73, P5, and P24. Conversely, the strains of *S. bacillaris*, *H. uvarum*, and *M. pulcherrima* appeared to be more tolerant to this compound. On the other hand, *T. delbrueckii* presented a specific profile, as growth was only affected above 750 mg/L, but they exhibited the highest growth inhibition at 1000 mg/L. The effects of Ser on the relative OD max of the strains showed a profile similar to GT (Supplementary Figure S2).

For most strains, the addition of TrpOH caused a decrease in growth in a dose-dependent manner (see **Figure 3A** and Supplementary Figure S2). The presence of TrpOH had a greater impact on GT than on maximal growth (**Figure 3B**), particularly in non-*Saccharomyces* strains (**Figure 3C**). Among them, the most tolerant strain was *M. pulcherrima* Mpp, which was only slightly affected at high doses of TrpOH. Conversely, the other *M. pulcherrima* strain, FLAVIA, was one of the most heavily affected, indicating that sensitivity to TrpOH is strain-dependent. In general, the *S. cerevisiae* strains were less affected by TrpOH.

Trpm influenced differently the growth of yeast strains, resulting in increases in the lag phase or in the GT, decreases in the OD max, and even no inhibitory effects at all (see two examples in **Figure 4A**). Thus, within the same species, we observed different responses to the presence of Trpm. For example, among *S. cerevisiae* strains, Vin7 only showed an increase during the lag phase, and there were no significant effects on the other growth parameters; QA23 primarily increased its GT and decreased the OD max, while the other *S. cerevisiae* strains were barely affected by Trpm (**Figures 4B,C** and Supplementary Figure S2). On the other hand, non-*Saccharomyces* strains were more affected by the presence of this biogenic amine, even at low concentrations, modifying all the growth parameters. Interestingly, in *M. pulcherrima* strains, the effects of Trpm on the OD max and GT were not dose-dependent, demonstrating similar inhibition from 100 to 1000 mg/L (**Figure 4** and Supplementary Figure S2).

### Effects of Culture Medium Composition on Filamentous Growth in Non-*Saccharomyce*s Species

**FIGURE 5:**
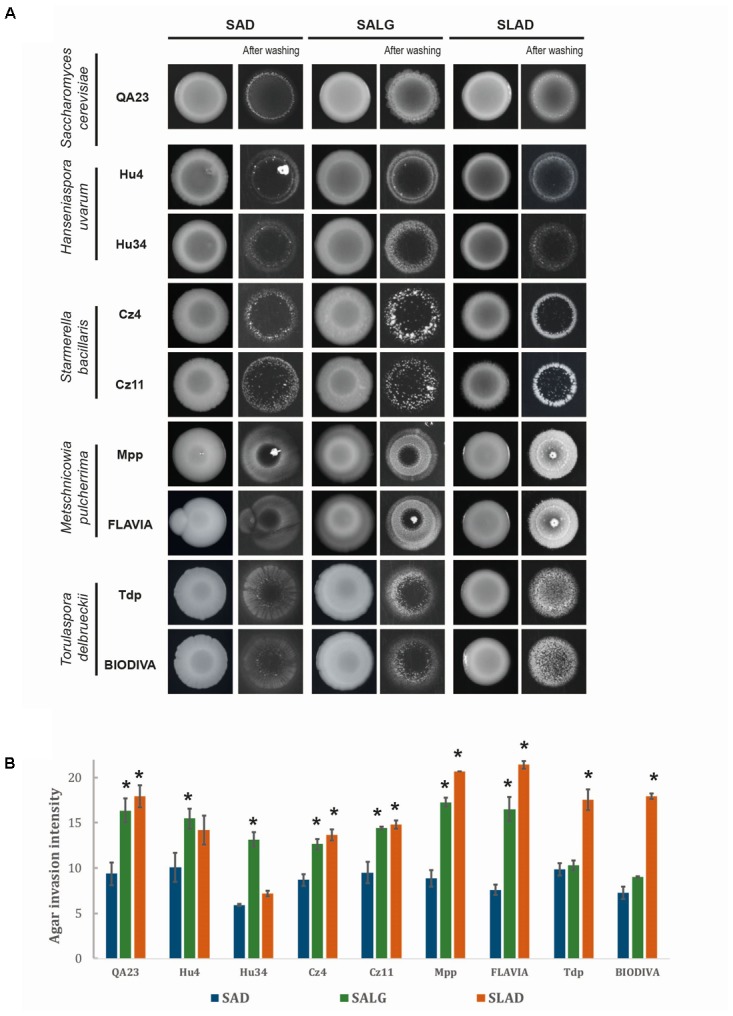
Invasive growth phenotypes of different wine yeast species. **(A)** In a plate washing assay (PWA), equal concentrations of cells were spread on media with different nutrient contents and incubated for 5 days at 28°C. **(B)** Quantification of invasive growth was performed after washing the plate via densitometry analysis. Cells were spotted in triplicate, and the average values are shown. Statistical analysis was carried out by comparing each strain with respect to rich media (SAD), using Tukey’s test statistical method; asterisk denotes a *p*-value < 0.05.

The aromatic alcohols and ethanol have been described as molecules signaling morphological changes in different yeasts, primarily in *S. cerevisiae* and *C. albicans*; therefore, we analyzed their effects on the non-*Saccharomyces* strains. We first studied invasive growth on rich (SAD) and nutrient-limiting [glucose (SALG) and nitrogen (SLAD)] media for all strains using *S. cerevisiae* QA23 as a control (**Figure 5**). Interestingly, all strains exhibited a certain degree of invasive growth. Moreover, media limited for glucose or nitrogen resulted in enhanced invasive growth for most of them. Specifically, on SLAD plates, most strains showed invasive growth that was significantly higher than on SAD, with the exception of *H. uvarum* strains. *M. pulcherrima* and *T. delbrueckii* strains were the most invasive non-*Saccharomyces* yeasts in the absence of nitrogen. Carbon source limitation (SALG) had a similar effect as nitrogen; most strains presented significant invasive growth compared to rich media, with the exception of the two *T. delbrueckii* strains.

**FIGURE 6:**
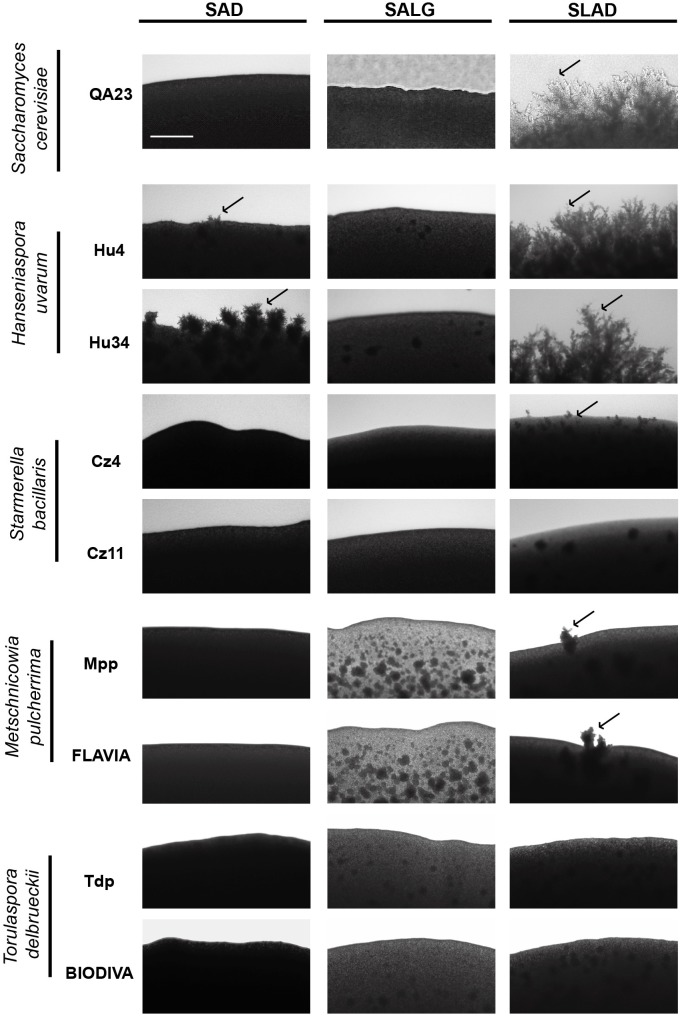
Pseudohyphal growth phenotypes of different wine yeast species. Cells were spotted on rich medium (SAD) and nutrient limitation media (SALG and SLAD). Colony peripheries were photographed after incubation for 5 days at 28°C. Scale bar is 50 μm. Arrows mark examples of pseudohyphae.

We also determined the ability of these yeasts to form pseudohyphae by analyzing the morphology of their colonies on SAD, SLAD, and SALG media. **Figure 6** shows the morphology of the colony peripheries at day 7. *H. uvarum* strains exerted the highest pseudohyphal phenotype, mainly in limitation of nitrogen (SLAD), similarly to the control strain. Surprisingly, these *H. uvarum* strains were also able to produce pseudohyphae on rich media. *M. pulcherrima* and *S. bacillaris* strains formed few filaments only in SLAD medium, and none of the tested strains underwent pseudohyphae in SALG medium. Thus, the lack of glucose was not a limiting factor to trigger this aspect of the filamentous growth response in non-*Saccharomyces* yeast.

### Effects of Alcohols on Filamentous Growth in Non-*Saccharomyces* Species

**FIGURE 7:**
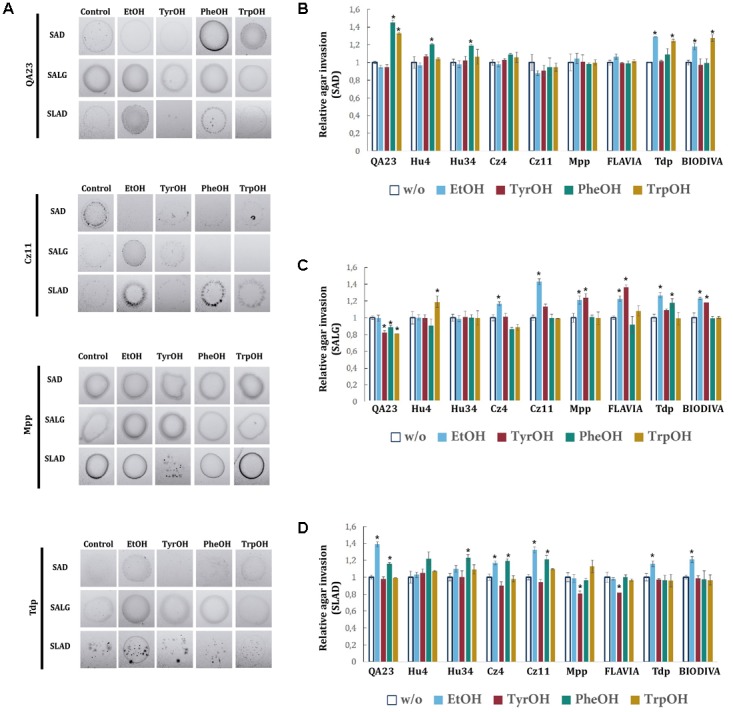
Invasive growth of wine yeast species in the presence of aromatic alcohols and ethanol. **(A)** In a plate washing assay (PWA), equal concentrations of cells were spread on SAD, SALG, and SLAD media in the presence of the aromatic alcohol (TyrOH, PheOH, or TrpOH) at 500 μM or 2% (v/v) EtOH and incubated for 3 days at 28°C. Panel **(A)** shows the results from the washed plate. The invasive growth obtained with different wine yeast species in SAD **(B)**, SALG **(C)**, and SLAD **(D)** was obtained via densitometry. Cells were spotted in triplicate, and the average agar invasion values were calculated. Relative invasion values were obtained by dividing the agar invasion in presence of each compound and the one of the controls (no-supplemented condition, w/o). Statistical analysis was performed comparing the effects of the alcohols in each strain relative to the control, *p*-value < 0.05.

The effects of alcohols on invasive growth were assayed on SAD, SALG, and SLAD plates, both with and without supplementation with different alcohols. In general, the effects of alcohols varied depending on the medium and the species (**Figure 7A**). On SAD medium (**Figure 7B**), TrpOH and PheOH promoted invasive growth in the *S. cerevisiae* strain. Among non-*Saccharomyces* species, PheOH only stimulated invasive growth in *H. uvarum*, while ethanol and TrpOH only in *T. delbrueckii*. Furthermore, no significant effects were observed in *S. bacillaris* or in *M. pulcherrima*. On SALG plates (**Figure 7C**), aromatic alcohols significantly decreased the invasive growth of the commercial QA23 strain. Among non-*Saccharomyces* strains, TrpOH and PheOH significantly promoted invasive growth on *H. uvarum* Hu4 and *T. delbrueckii* Tdp, respectively. Ethanol appeared to strengthen invasive growth in *S. bacillaris*, *M. pulcherrima*, and *T. delbrueckii*, while TyrOH presented similar effects in the two strains of *M. pulcherrima* and in the commercial *T. delbrueckii* BIODIVA strain. On SLAD plates (**Figure 7D**), ethanol induced invasive growth in the QA23 strain, as well as in both strains of *S. bacillaris* and *T. delbrueckii. H. uvarum* and *S. bacillaris* increased their invasive growth in the presence of PheOH. On the other hand, TyrOH significantly reduced the invasive growth of *M. pulcherrima* strains.

**FIGURE 8:**
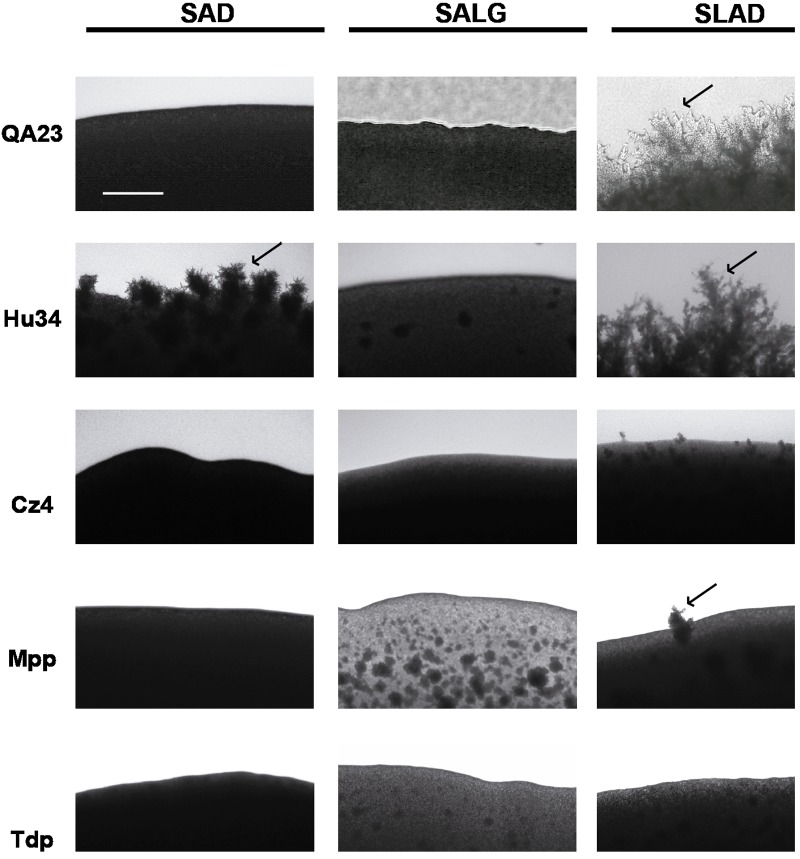
Pseudohyphal growth phenotypes of different wine yeast species in response to ethanol and aromatic alcohols. Cells were spotted on SLAD medium. Colony peripheries were photographed after incubation for 3 days at 28°C. Scale bar is 50 μm.

To study the effects of alcohols in pseudohyphal growth, we focused on SLAD medium (**Figure 8**). Ethanol and PheOH stimulated pseudohyphal formation in *S. cerevisiae*. However, the addition of alcohols to agar plates resulted in a reduction in filamentation in both strains of *H. uvarum*. Similar to *S. cerevisiae*, ethanol changed growth patterns to a more filamentous form in *S. bacillaris*, but the aromatic alcohols tested did not affect pseudohyphae development. TyrOH considerably increased filament formation in *M. pulcherrima*. Moreover, the two strains of *T. delbrueckii* tested did not form pseudohyphae when starved for nitrogen in the presence of any alcohol tested.

## Discussion

No organisms exist in isolation, all species share common environments and compete for nutrients. Interactions between organisms are commonplace and may be diverse. Although there are many examples of cooperation and symbiotic relationships among organisms, many interactions are combative, with one species profiting from another’s detriment. An excellent example of this is seen on rotting fruit, where yeast and other microorganisms compete for sugar food sources. Non-*Saccharomyces* yeasts are predominant in grape must, even during the first stages of spontaneous fermentations, but are rapidly replaced by *S. cerevisiae*, which completes the process (Fleet, 2003). Recently, some findings have associated interactions between species with the secretion of certain compounds by yeast during alcoholic fermentation (Ciani and Comitini, 2015; Wang et al., 2015a; Albergaria and Arneborg, 2016), such as some alcohols which are produced at high density by *S. cerevisiae* (Zupan et al., 2013). Our results showed that aromatic alcohols reduced yeast cell growth, especially in non-*Saccharomyces*, where the three fusel alcohols exerted negative effects on GT and maximal growth in most strains, even at low concentrations (100–250 mg/L). Instead, in *S. cerevisiae* strains, only TrpOH exhibited growth inhibition. These aromatic alcohols are produced by wine yeast and are found in alcoholic beverages at concentrations ranging from 4 to 197 mg/L PheOH, 100 to 450 mg/L TrpOH, and 5 to 40 mg/L TyrOH (Swiegers et al., 2005). Non-*Saccharomyces* strains are able to produce these aromatic alcohols, but at lower concentrations than *S. cerevisiae* (Zupan et al., 2013; González, 2017), however, the negative effects on the growth of these alcohols were more pronounced in non-*Saccharomyces*. Thus, the production of aromatic alcohols may play a role in certain yeast interactions, inhibiting the growth of non-*Saccharomyces* strains and even directing the replacement of these species during alcoholic fermentation by the major producer species, *S. cerevisiae*. Nevertheless, in this study, we tested the effects of these alcohols individually, but mixtures of them may have greater impact on yeast growth.

Mel is synthesized from tryptophan and exhibits various biological activities in humans, such as antioxidant activity (Reiter et al., 2001; Anisimov et al., 2006). It has been proved that yeasts generate low concentrations of Mel during alcoholic fermentation (Rodriguez-Naranjo et al., 2012); however, its role in yeast regulation is still unknown. In our study, the presence of Mel in the media did not affect the growth of the yeast strains tested. In contrast to Mel, its precursor, Ser, considerably reduced the maximal growth and doubling time of all strains tested, and was the most inhibiting compound tested, which indicates that Ser has toxic effects in yeast. Indeed, Ser has previously shown antifungal activity against *Candida* and *Aspergillus* spp. *in vitro* (Lass-Flörl et al., 2002, 2003). On the other hand, Trpm mostly affected the lag phase, being reduced at low concentrations but increased at high concentrations. Trpm levels in wines are usually very low (0.02–0.2 mg/l), and its synthesis largely depends on fermentation temperature but not on supplementation with its precursor amino acid (Lorenzo et al., 2017), Ser is found at very lower concentration at the end of alcoholic fermentation (Fernández-Cruz et al., 2017). Therefore, although Trpm and Ser appear to significantly affect different growth parameters, this does not occur at concentrations usually found in wines.

Recently, the death of non-*Saccharomyces* yeasts in mixed fermentation with *S. cerevisiae* was associated with mechanisms mediated through cell-to-cell contact as well as high cell densities (Nissen et al., 2003, 2004; Pérez-Nevado et al., 2006; Renault et al., 2013). However, the role of cell-to-cell communication through QSM in inhibiting the growth of certain yeast strains during mixed-culture fermentation remains unclear (Wang et al., 2015b; Avbelj et al., 2016). QS in yeasts involves a morphological transition from a filamentous to a yeast form, or *vice versa* (Sprague and Winans, 2006). Yeasts undergo this transition from a unicellular to a filamentous form in response to environmental cues, which may arise from alterations in nutrient concentrations or in the presence of auto-inductive molecules that are secreted by cells (Chen and Fink, 2006). Stimuli that trigger filamentous growth include nitrogen limitation (Gimeno et al., 1992) and glucose limitation (Cullen and Sprague, 2000). Filamentation is well established in *Saccharomyces* (Chen and Fink, 2006; Cullen and Sprague, 2012) and the dimorphic fungal human pathogen *C. albicans* (Hornby et al., 2001; Chen et al., 2004), but little is known about this type of growth in other genera and species of yeasts (Gori et al., 2011; Pu et al., 2014; van Rijswijck et al., 2015). In our study, we tested two strains each of the major genera involved during wine fermentation to test their ability to penetrate surfaces (invasive growth) or to form pseudohyphae. All strains tested were wild yeasts isolated from wine environments and were able to invade, even in rich media. Indeed, natural yeast isolates exhibit high levels of invasion (Casalone et al., 2005), allowing them to colonize natural niches, such as grapes. According to Pitoniak et al. (2009), yeasts require the filamentous growth pathway and Flo11 to be able to fully colonize this environment. Nutrient limitation also promotes increased invasive growth in non-*Saccharomyces* species. The *S. bacillaris* and *M. pulcherrima* strains increased their invasive growth both under glucose and nitrogen limitation, but they only formed small pseudohyphae with nitrogen limitation. Indeed, the ability to form pseudohyphae and invade agar upon nutrient deprivation provides a selective advantage to yeast cells, facilitating foraging for scarce nutrients at a distance from their initial position (Casalone et al., 2005). On the other hand, *H. uvarum* exhibited a striking behavior because its cells primarily invade the agar under glucose limitation but form a large number of pseudohyphae under nitrogen limitation and, to a lesser extent, in rich media. The ability of these strains to form pseudohyphae in rich media may be an advantage to colonize fruits by adhesion and a possible reason for the wide distribution of this species on natural fruit surfaces; in some studies, *H. uvarum* is the main species found in grape habitats (Pretorius, 2000; Beltran et al., 2002; Cadez et al., 2002; Ocón et al., 2010; Padilla et al., 2016), Finally, *T. delbrueckii* was the only species that did not form pseudohyphae in any of the tested media. Nevertheless, this species was able to invade under nitrogen limitation. This suggests the differential regulation of both phenotypes in this species. A possible explanation for this lack of pseudohyphal growth may be related to its ability to flocculate in liquid medium, especially in YPD medium. Both phenotypic traits are mediated by the same family gene and a recent study demonstrated that variations in the amino acid sequence of the adhesion domain of Flo11 causes different flocculation activities (Barua et al., 2016).

Overall, the two strains of each species tested presented similar behaviors, indicating that filamentous growth is a similar trait in several species. Aromatic alcohols have been reported to possess QS activity, and their effects together with ethanol on *S. cerevisiae* morphology have been thoroughly described (Chen and Fink, 2006; González et al., 2017). In this study, the effects of aromatic alcohols and ethanol were analyzed in three different media, which differed in their glucose and ammonium content. As previously described, PheOH and TrpOH exerted effects on filamentous growth in *S. cerevisiae*. However, these results are not completely in concordance with Chen and Fink (2006), since they observed that PheOH and TrpOH both exerted effects on pseudohyphal growth but only PheOH affected invasive growth, and in our study we observed the opposite. Moreover, we also observed inhibitory effects on pseudohyphae with all aromatic alcohols in low glucose medium. In *H. uvarum*, the sole aromatic alcohol that promoted invasive growth was PheOH, both in rich and in nitrogen-limiting media. A reduction in pseudohyphae formation was observed in the presence of aromatic alcohols, which also occurred with farnesol in *C. albicans* (Hornby et al., 2001). In a recent study, Pu et al. (2014) described the involvement of PheOH in filamentous growth, adhesion, and biofilm formation in *H. uvarum*. On the other hand, TyrOH has been described as an inducer of filamentous growth in *C. albicans* (Chen et al., 2004). However, TyrOH did not affect significantly *S. bacillaris* growth in any of the conditions tested, as it might be expected due to its greater proximity to *C. albicans*. Anyway, this species produced very low concentration of aromatic alcohols, even in a previous study no synthesis was detected (Zupan et al., 2013; González, 2017). Therefore, in this species, other molecules may be the signals that initiate changes in morphogenesis, similar to *C. albicans* with farnesol (Kruppa, 2009). The effects of TyrOH on morphological changes were also observed in *M. pulcherrima*, suggesting a possible signaling role also in this species. Ethanol has been extensively reported to stimulate pseudohyphal growth in *S. cerevisiae* (Lorenz et al., 2000; González et al., 2017). In our study, ethanol affected all species to varying degrees, with the exception of *T. delbrueckii*. However, even in this species, ethanol promoted invasive growth under all tested conditions. As we have previously shown, *T. delbrueckii* did not undergo pseudohyphal growth under any of the tested conditions, but these strains presented flocculent growth in liquid media, which may suppress filamentation, as both responses are controlled by the same gene family (Soares, 2011).

Therefore, the aromatic alcohols appear to be species-specific signaling molecules because different species manifest different responses to these auto-regulatory molecules. This finding was previously observed for *S.cerevisiae* and *C.albicans*: Chen and Fink (2006) demonstrated that different aromatic alcohols exert different effects on the morphogenesis of these two yeast species.

## Conclusion

We demonstrated that aromatic amino acid-derived compounds produced during alcoholic fermentation by yeast, and at the concentrations found in fermented beverages, modulate the growth of certain yeast species. Among these compounds, aromatic alcohols appear to be the most interesting because yeasts synthesize these compounds at levels that have physiological effects, suggesting a possible role in microbial interaction during wine fermentation. Our study reinforces the idea that these molecules play roles as QSM on both *Saccharomyces* and non-*Saccharomyces* species, as they appear to be able to induce or repress their filamentous and vegetative growth.

## Author Contributions

BG designed and performed the experiments, analyzed and discussed the results, and wrote the manuscript. JV performed the experiments and analyzed and discussed the results. PC designed the experiments, discussed the results, and wrote the manuscript. AM, M-JT, and GB designed the experiments, analyzed and discussed the results, wrote the manuscript, and rose the funding.

## Conflict of Interest Statement

The authors declare that the research was conducted in the absence of any commercial or financial relationships that could be construed as a potential conflict of interest.

## Footnotes

1 http://rsb.info.nih.gov/ij/
