# Supplementary material for: Aromatic Amino Acid-Derived Compounds Induce Morphological Changes and Modulate the Cell Growth of Wine Yeast Species
Source: Front Microbiol. 2018 Apr 11;9:670. doi: 10.3389/fmicb.2018.00670 (PMC5904269; doi:10.3389/fmicb.2018.00670)

frontiers

Supplementary Material

Aromatic amino acid-derived compounds induce morphological changes and modulate the cell growth of wine yeast species

Beatriz González [1], Jennifer Vázquez [1], Paul J. Cullen [2], Albert Mas [1], Gemma Beltran [1]*, and María Jesús Torija [1]

*** Correspondence:** Corresponding Author: gemma.beltran@urv.cat

Supplementary Figure 1. Bi-plots of Principal component analysis (PCA) using the following variables: maximal cell density (OD) and generation time (Gt) obtained by the supplementation of each compound at 10 mM (NH4)2SO4 (A) and 1 mM (NH4)2SO4 (B).

A

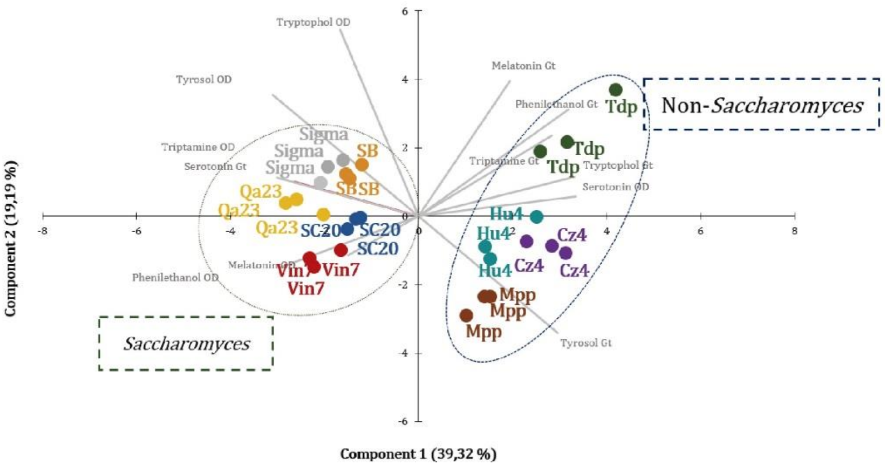

B

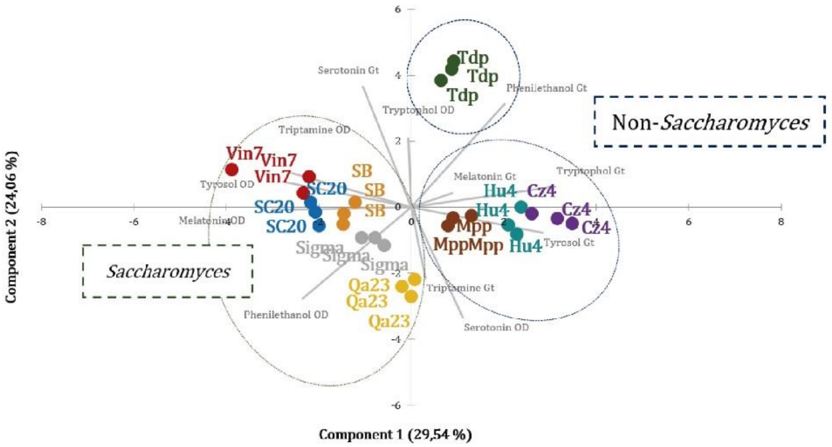

Supplementary Figure 2. Effects of increasing compound, Ser (A), TrpOH (B), Trpm (C) concentrations on yeast growth. Different compounds were added to minimal medium (1 mM $(NH_4)_2SO_4$) at increasing concentrations (50, 100, 250, 500, 750 and 1000 ppm). Statistical analysis was performed comparing the effects of compound concentrations in each strain; p-value <0,05. The fold-change for each growth parameter was determined in relation to the control (without compounds).

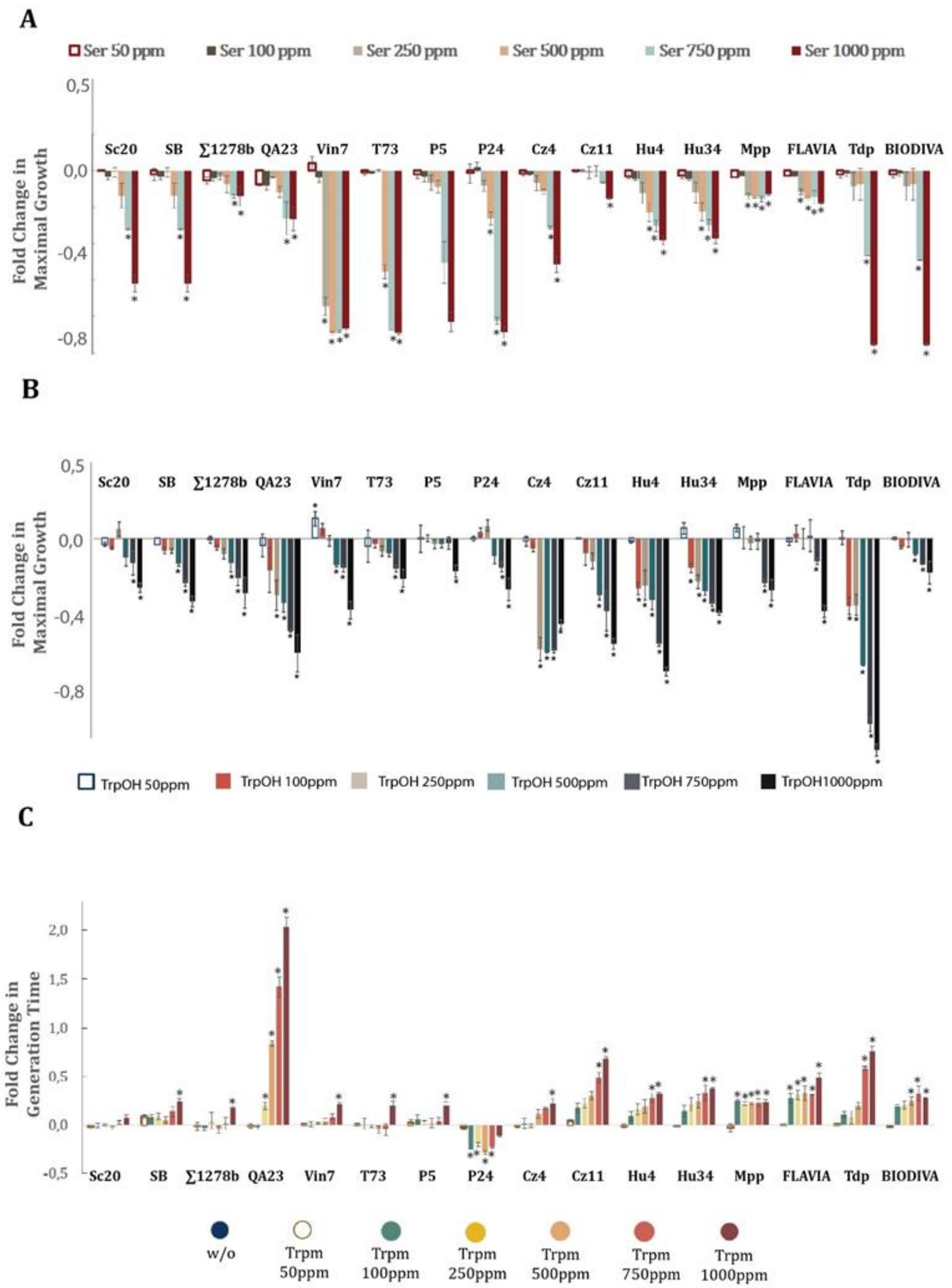

Supplementary Figure 3. Effects of increasing aromatic alcohols, TyrOH (A) and PheOH (B), concentrations on yeast growth. Different compounds were added to minimal medium (1 mM $(NH_4)_2SO_4$) at increasing concentrations (50, 100, 250, 500, 750 and 1000 ppm). Statistical analysis was performed comparing the effects of compound concentrations in each strain; p-value <0,05. The fold-change for each growth parameter was determined in relation to the control (without compounds).

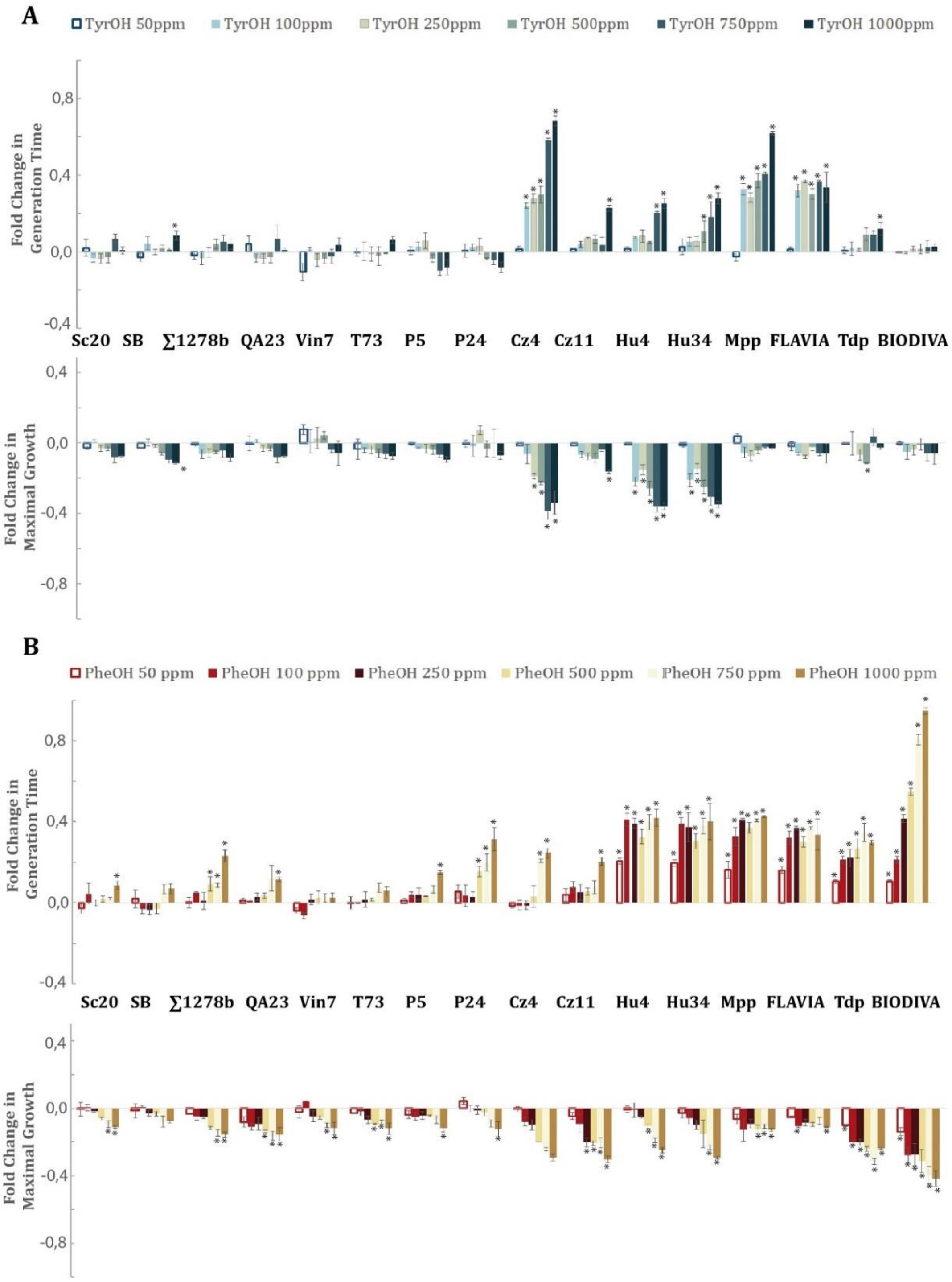

Supplement: Supplementary file 2 [file Data_Sheet_1.PDF]
